# Swin Attention Augmented Residual Network: a fine-grained pest image recognition method

**DOI:** 10.3389/fpls.2025.1619551

**Published:** 2025-06-19

**Authors:** Xiang Wang, Zhiyong Xiao, Zhaohong Deng

**Affiliations:** School of Artificial Intelligence and Computer Science, Jiangnan University, Wuxi, China

**Keywords:** artificial intelligence, deep learning, fine-grained insect image, Swin Transformer, image classification

## Abstract

Pest infestation is a major cause of crop losses and a significant factor contributing to agricultural economic damage. Accurate identification of pests is therefore critical to ensuring crop safety. However, existing pest recognition methods often struggle to distinguish fine-grained visual differences between pest species and are susceptible to background interference from crops and environments. To address these challenges, we propose an improved pest identification method based on the Swin Transformer architecture, named Swin-AARNet (Attention Augmented Residual Network). Our method achieves efficient and accurate pest recognition. On the one hand, Swin-AARNet enhances local key features and establishes a feature complementation mechanism, thereby improving the extraction capability of local features. On the other hand, it integrates multi-scale information to effectively alleviate the problem of fine-grained feature ambiguity or loss. Furthermore, Swin-AARNet attained a classification accuracy of 78.77% on IP102, the largest publicly available pest dataset to date. To further validate its effectiveness and generalization ability, we conducted additional training and evaluation on the citrus benchmark dataset CPB and Li, achieving impressive accuracies of 82.17% and 99.48%, respectively. SwinAARNet demonstrates strong capability in distinguishing pests with highly similar appearances while remaining robust against complex and variable backgrounds. This makes it a promising tool for enhancing agricultural safety management, including crop environment monitoring and early invasion warning. Compared with other state-of-the-art models, our proposed method exhibits superior performance in pest image classification tasks, highlighting its potential for real-world agricultural applications.

## 1 Introduction

Pests and diseases pose a significant threat to global agriculture, resulting in substantial economic losses. As one of the primary challenges in agricultural production, pest infestations lead to severe yield reductions and facilitate the spread of crop diseases. For example, the Greening (Diaphorina citri), also called Huanglongbing (HLB) alone caused $13.2 billion in damage to Florida between 2005 and 2016 (Court et al., 2018). Currently, most pest control strategies rely on pesticide spraying and crop isolation. Although these methods can reduce the damage caused by pest infestations to some extent, they have led to increasingly serious problems such as environmental pollution, food safety concerns, and ecological degradation. Therefore, to achieve modernized and scientific agricultural production, existing pest detection and control strategies urgently need improvement.

**Figure 1:**
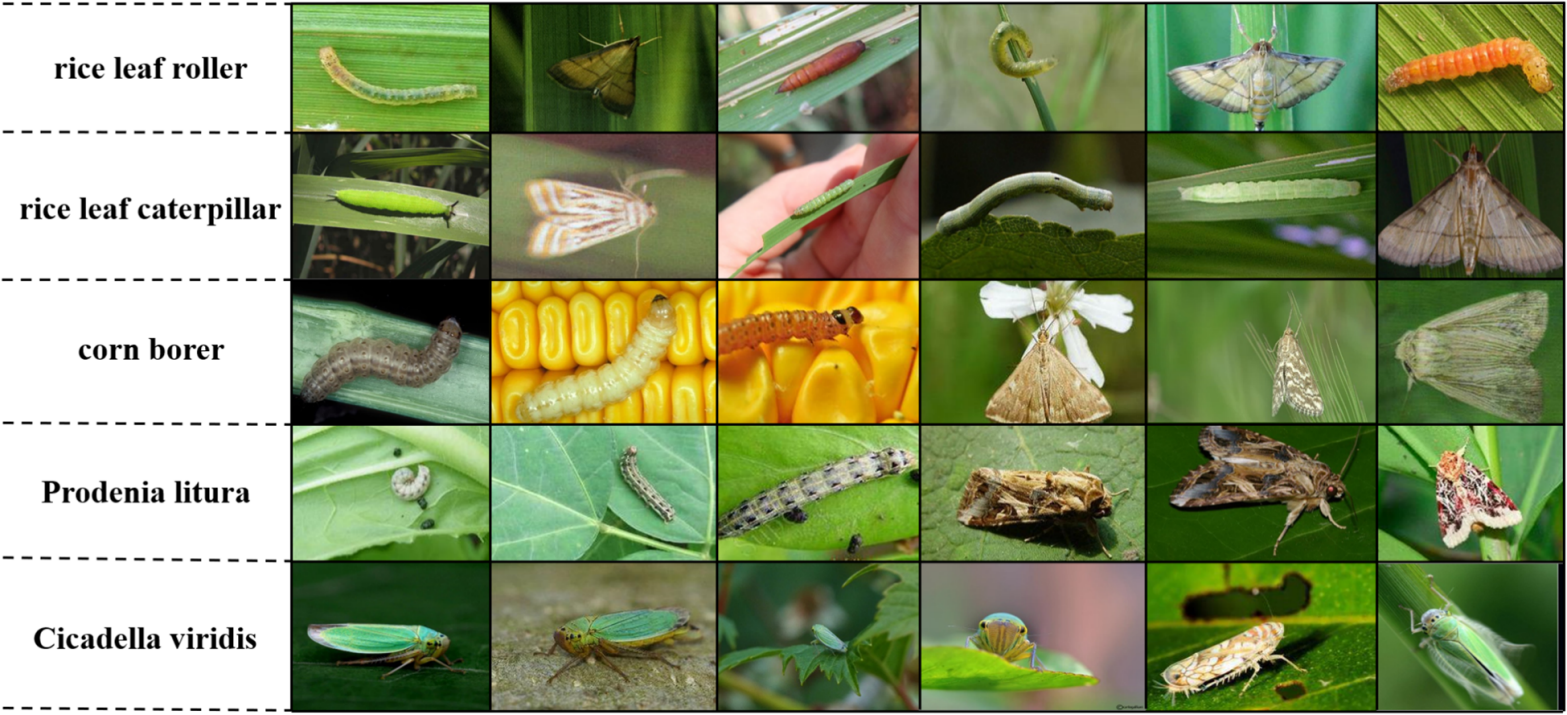
Example of fine-grained insect classes from IP-102 dataset. Among them, rice leaf roller, rice leaf caterpillar, corn borer, Prodenia litura, and Cicadella viridis are images of some categories captured from IP102. The morphology of their larvae and adults varies greatly. And the morphology of adult insects of different categories is also similar.

Despite the convenience brought by Internet technologies in daily life, their application in specific domains still has substantial limitations. Taking the rice leaf roller as an example (as shown in the first row of **Figure 1**), when users attempt to search for pest-related information using search engines, they are often confronted with numerous irrelevant results, many of which are misleading. Moreover, when users are unable to identify the exact name of a pest, the search engine cannot perform image-based category retrieval. This highlights a key limitation of Internet technologies in domain-specific applications. In practice, the content indexed by search engines largely depends on manually uploaded data. Since human annotations inherently possess subjectivity and limitations, the accuracy and reliability of the search results are often compromised. As a result, current technologies are unable to provide high-precision category classification and information management for pests. To address this challenge, it is essential to develop efficient fine-grained image analysis algorithms tailored for pest classification, in order to meet the demands of intelligent agricultural monitoring and ensure sustainable agricultural development.

Previous classification systems primarily relied on handcrafted and feature-based methods. Manual approaches, such as SIFT (ScaleInvariant Feature Transform) (Lowe, 2004) and HOG (histograms of oriented gradient) (Dalal and Triggs, 2005), performed well in representing low-level features, such as color and texture. However, these methods typically extracted a limited set of distinctive features to represent pests, making it difficult to evaluate them on complex datasets with diverse characteristics. For different types of pests, manual inspection of features proved inefficient, time-consuming and lacked the ability to capture high-level semantic information. In recent years, mobile technologies have been widely adopted to perform various tasks in the agricultural sector. Under such circumstances, the integration of artificial intelligence to replace manual processing in classification tasks has become feasible. This shift offers significant potential to address challenges such as labor shortages and low recognition efficiency, thereby providing substantial practical value and application prospects. Numerous researchers have published extensive studies in this area and have implemented these technologies in real-world pest recognition systems. Previously, traditional machine learning models, such as support vector machines (SVMs), neural networks, decision trees, and k-nearest neighbors, have been utilized to accurately process pest images (Júnior and Rieder, 2020; Martineau et al., 2017). For example, Ebrahimi et al. (2017) developed an image processing program to recognize pests in greenhouse environments. They employed a Support Vector Machine (SVM) approach and successfully detected targets such as thrips with an error rate of less than 2.5%. However, applying such methods to image classification tasks still presents several challenges, particularly when dealing with insects that exhibit similar appearances but belong to different categories. Fortunately, Convolutional Neural Networks (CNNs) have demonstrated superior performance over traditional machine learning methods in visual tasks, offering better generalization capabilities when handling complex problems in pest recognition. Currently, CNNs have been widely applied to various agricultural tasks. For instance, Thenmozhi and Reddy (2019) proposed an efficient deep CNN model designed to assist farmers in accurately classifying and recognizing pest larvae. Their model was evaluated on three publicly available insect datasets—NBAIR, Xie1, and Xie2—for species-level classification. The results demonstrated that the proposed CNN model outperformed several pre-trained deep learning architectures, including AlexNet, ResNet (Residual Neural Network), and GoogLeNet, achieving maximum classification accuracies of 96.75%, 97.47%, and 95.97%, respectively. This highlights the strong potential of deep CNNs for accurate pest recognition in practical agricultural scenarios. However, CNNs primarily excel at capturing local features, while exhibiting certain limitations in extracting global representations. Additionally, their performance is often influenced by image resolution, which can lead to the loss of fine-grained information in some cases. These constraints pose challenges when applying CNNs to large-scale pest recognition tasks, highlighting the need for further research and architectural improvements to enhance their effectiveness in such complex scenarios.

The Vision Transformer (ViT) proposed by Dosovitskiy (Dosovitskiy, 2020) addresses challenges in multiclass image classification and high shape similarity among classes, achieving strong classification performance across a range of vision tasks. Researchers have tried to perform some classification tasks for pests based on ViT models. Liu et al. (2022a) proposed a feature relationship conditional filtering (FRCF) based on k-NN graph to conditionally filter different relevant data from the source domain and generate a subset of the source domain, which is more effective than CNN-based methods in the field of pest and disease classification. Currently, both CNNs and Transformers have demonstrated outstanding performance in image-related tasks across multiple domains, including medical imaging (Xiao et al., 2022; Ji et al., 2023; Xiao et al., 2024b) and food image analysis (Gao et al., 2024; Xiao et al., 2025a). Peng and Wang (2022) proposed a scalable architecture that combines CNNs and Transformers, achieving a better trade-off between network parameters and accuracy. This work offers new insights into the ecological management of pests and diseases and provides valuable implications for future research in eco-informatics. However, despite overcoming some of the limitations of CNNs, these methods still face several challenges when deployed in real-world agricultural environments. These challenges are summarized as follows:

1. These networks typically focus on global windows, which may overlook subtle visual differences between pest species. For instance, various species of moths often exhibit highly similar appearances, and more critically, their larvae are nearly indistinguishable, making accurate recognition extremely difficult.
2. In real agricultural scenarios, pest images are often affected by factors such as lighting conditions, variations in pest size and color, occlusions, and the background color of crops. Therefore, an approach capable of enhancing local feature representations is urgently needed.

Motivated by these challenges, this study proposes a novel pest recognition architecture based on deep learning, incorporating two key components: DWAblock (Depth-wise separable residual attention block) and GSA (Global Spatial Attention). Through the effective integration of these components, the proposed method can efficiently extract local features from pest images while exhibiting strong robustness against environmental interference. The main contributions of this paper are summarized as follows:

1. A more efficient pest recognition model is designed within a deep learning framework;
2. The proposed DWAblock extracts localized channel feature representations, addressing the challenge of environmental disturbances in complex settings;
3. The proposed GSA effectively fuses multi-scale information while preserving channel features, thus mitigating the issue of fine-grained feature ambiguity or loss.

In the era of agricultural modernization, the frequent occurrence of agricultural disasters and abnormal global climate changes have severely constrained agricultural development. Among these challenges, the increasing diversity of pest species has increased the complexity of crop pest control, resulting in significant agricultural losses. Accurate identification of pest morphology and texture characteristics is central to addressing pest recognition challenges. Swin-AARNet is designed to mine and enhance local feature representations, integrate these representations deeply, and ultimately extract and recognize them through a spatial feature extractor. The goal of Swin-AARNet is to facilitate more effective pest detection, address manual classification inefficiencies, and enable timely and effective pest control. By minimizing the likelihood of pest outbreaks, the model has substantial research value.

## 2 Related work

### 2.1 Pest classification

Deep learning-based recognition models have been widely used in agriculture (Tannous et al., 2023; Sun et al., 2024). Additionally, as shown in **Table 1**, For instance, Hechen et al. (2024) developed a novel Dilated-Windows-based Vision Transformer with Efficient-Suppressive-self-attention architecture, named DWViT-ES, which employs a suppression attention architecture. This approach reduced the model parameters and computational complexity while expanding the receptive field of local attention. It achieved top-1 accuracy rates of 76.0% and 78.7% on the IP102 and CPB datasets, respectively. Furthermore, Wei et al. (2024) introduced an improved version of the YOLOv8n model, termed AEC-YOLOv8n, which enhanced the representation of features by capturing contextual similarities and differences and facilitating the exchange of information between features. Nguyen et al. (2024) proposed a novel pest classification learning assistant tool DeWi. By refining several Convolutional Neural Networks, the DeWi model effectively learned the deep features of pests and generalized well across a wide range of insect categories. It achieved the highest performance on two pest datasets, IP102 and D0, with accuracies of 76.44% and 99.79%, respectively. Ayan (2024) utilized a hyperparameter optimization strategy based on a genetic algorithm, testing three different CNN models (MobileNetV2, DenseNet121, and InceptionResNetV2) across three insect datasets. This method achieved accuracies of 71.84%, 99.89%, and 97.58% on the IP102, D0, and Deng datasets, respectively. Finally, Ren et al. (2019) used residual feature reuse blocks to construct a feature reuse residual network (FR-ResNet) and evaluated it on the IP102 benchmark data set. The experimental results indicate that FR-ResNet shows strong adaptability and achieves significant performance improvements in pest classification. Qian et al. (2025) proposed a parallel architecture composed of a feature fusion module (FFM) and a mixed attention module (MAM), which effectively balances fine-grained feature extraction and improves pest recognition accuracy in complex scenarios. This architecture achieved evaluation accuracies of 75.74%, 99.82%, and 98.77% on the IP102, D0, and Li datasets, respectively. Lv et al. (2024) constructed the rice pest and disease dataset (RPDD) and expanded the dataset through data augmentation techniques. They proposed a Lightweight Multi-scale Feature Extraction Network (LMN), which achieved an average classification accuracy of 95.38% on the RPDD dataset. These results clearly demonstrate the feasibility of applying deep learning techniques to fine-grained pest recognition. However, the high-precision recognition capability of models under complex real-world conditions still requires further improvement.

**Table 1:** Classification methods used in insect recognition studies.

Year	References	Datasets	Methods
2019	Ren et al. (2019)	IP102	FR-ResNet.
2024	Nguyen et al. (2024)	IP102 and D0	DeWi.
2024	Hechen et al. (2024)	IP102 and CPB	DWViT-ES.
2024	Wei et al. (2024)	IP102	AEC-YOLOv8n.
2024	Ayan (2024)	IP102, D0 and Deng	MobileNetV2, DenseNet121 and InceptionResNetV2.
2024	Lv et al. (2024)	RPDD	LMN.
2025	Qian et al. (2025)	IP102 and D0 and Li	FFM and MAM.

### 2.2 Fine-grained visual classification

Fine-Grained Visual Classification (FGVC) aims to distinguish subcategories that belong to the same high-level category but exhibit subtle inter-class differences—for example, different pest species within the class Insecta. Since the differences among subcategories often appear only in small, localized regions, FGVC presents significant challenges in feature extraction and discrimination, relying heavily on effective modeling of local discriminative features. In recent years, substantial progress has been made in this field. Zheng et al. (2017) proposed a multi-attention convolutional neural network (MA-CNN), which consists of convolutional and grouped channel classification subnetworks. They also introduced two loss functions to guide the multi-task learning of these two subnetworks. MA-CNN achieved state-of-the-art performance on several benchmark datasets, including CUB-Birds, FGVC-Aircraft, and Stanford-Cars. Ding et al. (2021) proposed the Attention Pyramid Convolutional Neural Network (AP-CNN), which incorporates a top-down feature pathway and a bottom-up attention pathway. This dual-path optimization strategy not only enhances local region features but also suppresses background noise, leading to improved feature representation. AP-CNN achieved superior performance on the CUB-200-2011, FGVC-Aircraft, and Stanford-Cars datasets. Song and Yang (2021) designed a feature boosting and suppression module to capture the most salient parts of the feature maps. Additionally, they introduced a feature diversification module to learn semantically complementary information. To address the challenge of distinguishing between highly confusing classes with subtle differences, Zhuang et al. (2020) proposed a simple but effective Attentive Pairwise Interaction Network (API-Net). This method identifies fine-grained image pairs through iterative interaction. API-Net outperformed previous state-of-the-art methods, achieving accuracies of 90.0%, 93.9%, 95.3%, 90.3%, and 88.1% on the CUB-200-2011, Aircraft, Stanford Cars, Stanford Dogs, and NABirds datasets, respectively.

Meanwhile, in the domain of fine-grained pest image recognition, researchers have conducted extensive and targeted research. Jin et al. (2021) proposed a Multi-Stream Aggregation Network (MSA-NET) for fine-grained pest and disease identification. The model integrates three mainstream architectures, ResNet, NTS-Net (Navigator-Teacher-Scrutiniser Network) and FAST-MPN-COV (Towards Faster Training of Global Covariance Pooling Network) by constructing a multi-stream feature extractor by constructing a multi-stream feature extractor. A fusion module based on NetVLAD (Network Vector of Locally Aggregated Descriptors) is introduced to effectively aggregate diverse feature representations, providing a high-efficiency recognition framework for pest and disease classification. Linfeng et al. (2023) introduced a novel convolutional neural network-based model (MSSN) that combines attention mechanisms, feature pyramids, and fine-grained modeling. The model achieved a maximum accuracy of 86.35% on large-scale datasets such as the Large-Scale Pest Dataset. Ma et al. (2021) developed a fine-grained identification model of pests based on probability fusion network (FPNT), achieving an average processing time of 61 ms and an average recognition accuracy of 93.18% across multiple benchmark comparisons, offering a practical reference for pest and disease prevention and early warning systems. Lin et al. (2023) proposed a fine-grained pest identification method based on a graph pyramid attention, convolutional neural network (GPA-Net), aimed at supporting modern intelligent agriculture and environmental protection. GPA-Net achieved top accuracies of 99.0%, 97.0%, and 56.9% on the Cassava Leaf, AI Challenger, and IP102 pest datasets, respectively, demonstrating strong capabilities in pest and disease differentiation. In addition, Zhang et al. (2023a) developed a multimodal fine-grained transformer (MMFGT) model, enhancing the Transformer architecture to further boost pest recognition performance. MMFGT achieved a recognition accuracy of 98.12% across multiple strong baselines, showcasing its great potential in fine-grained pest image classification.

### 2.3 Transfer learning

Transfer learning (Yosinski et al., 2014) is a strategy that uses knowledge acquired from one task as a foundation to perform a separate yet related task. In recent years, transfer learning has achieved groundbreaking progress in deep learning, showing immense potential, particularly in scenarios with limited computational resources or scarce data. The core idea is to transfer the knowledge learned from a model trained in an existing task or domain to another related task, thus improving the efficiency and performance of learning for the new task. Currently, transfer learning has been widely applied in domains such as natural language processing, computer vision, and medical imaging.

The most common approach to transfer learning is the pre-training and fine-tuning strategy, which has become a mainstream paradigm, particularly prominent in Natural Language Processing (NLP). Fine-tuning, a core aspect of transfer learning, has emerged as a notably faster and more accurate method compared to building models from scratch (Toscano-Miranda et al., 2024). This approach leverages pre-trained models, adapting them to specific pest recognition tasks, thereby streamlining the process and enhancing accuracy in agricultural pest management (Zhang et al., 2023b). Devlin (2018) introduced the BERT (Bidirectional Encoder Representation from Transformers) model, which involved pre-training on largescale unsupervised text data followed by supervised fine-tuning on specific tasks (e.g., text classification, named entity recognition). This approach significantly improved task performance and marked a milestone in the field of NLP. Furthermore, the RoBERTa (a Robustly Optimized BERT Pretraining Approach) model (Liu, 2019) improved the training process by using larger datasets, extended training durations, and optimized unsupervised task designs. This not only improved the effectiveness of pre-trained models but also exhibited exceptional transfer capabilities on downstream tasks. Compared to earlier versions of BERT, the success of RoBERTa further validated the powerful ability of transfer learning to capture general language representations through pre-trained models.

Cross-domain transfer learning has also been highly impactful in the field of computer vision. He et al. (2016) demonstrated that the ResNet architecture, pre-trained on large-scale datasets such as ImageNet, could be transferred to various domain-specific tasks (e.g., medical image analysis), proving that pre-trained models maintain strong generalization capabilities even on small-sample datasets. At the same time, Vision Transformer (Dosovitskiy, 2020) introduced attention mechanisms in visual tasks, significantly improving transfer capabilities between different domain tasks. Meanwhile, the combination of self-supervised learning and transfer learning has emerged as one of the most cutting-edge research areas since 2021. He et al. (2022) proposed the MAE model (Masked Autoencoders), which, based on self-supervised learning, pre-trains masked image models on unlabeled data to capture rich visual representations. These representations can then be transferred to downstream tasks such as image classification and object detection. The MAE study demonstrated that combining self-supervised pre-training with transfer learning enables effective transfer performance even in data-scarce scenarios, breaking the traditional dependency of transfer learning on large amounts of labeled data.

Moreover, cross-domain and cross-modal transfer learning has emerged as a key research direction in recent years. Zolfaghari et al. (2021) investigated cross-domain transfer between visual and language modalities, proposing a cross-modal contrastive learning method that jointly learns from visual and linguistic information to improve the model’s generalization ability.

## 3 Materials and methods

### 3.1 Swin Transformer

The Swin Transformer, introduced by Liu et al. (2021b), is a novel visual feature transformer that produces a hierarchical feature representation. Its primary feature extraction mechanism relies on shifted window calculations, where self-attention is confined within non-overlapping local windows while allowing cross-window connections, leading to improved efficiency. This hierarchical architecture enables flexible multi-scale modeling and achieves linear computational complexity relative to image size.

These characteristics make the Swin Transformer highly compatible with a wide range of visual tasks, including image classification and semantic segmentation. With the rise of computer vision, various Swin Transformer variants have emerged. Xiao et al. (2024a) proposed a new DRConvBlock (depthwise separable residual convolutional blocks) model and MLP-GD (multi-layer perceptron based on global average pooling and dropout), based on the Swin Transformer architecture. These models address the challenge of Swin Transformer in fine-grained recognition, particularly in accurately distinguishing shape differences in food items that belong to the same category. Similarly, Xiao et al. (2025b) developed a high-precision food image recognition method (FoodCSwin) based on the CSwin network for dietary assessment. The proposed method innovatively integrates the DiffAugment data augmentation strategy with a Local Feature Dual Branch Enhancement Block (LFDB-Block), enabling effective discrimination between nutritionally similar but visually distinct food items within the same category. This approach provides more accurate technical support for dietary nutrition assessment.

### 3.2 General structure of the methodology

**Figure 2:**
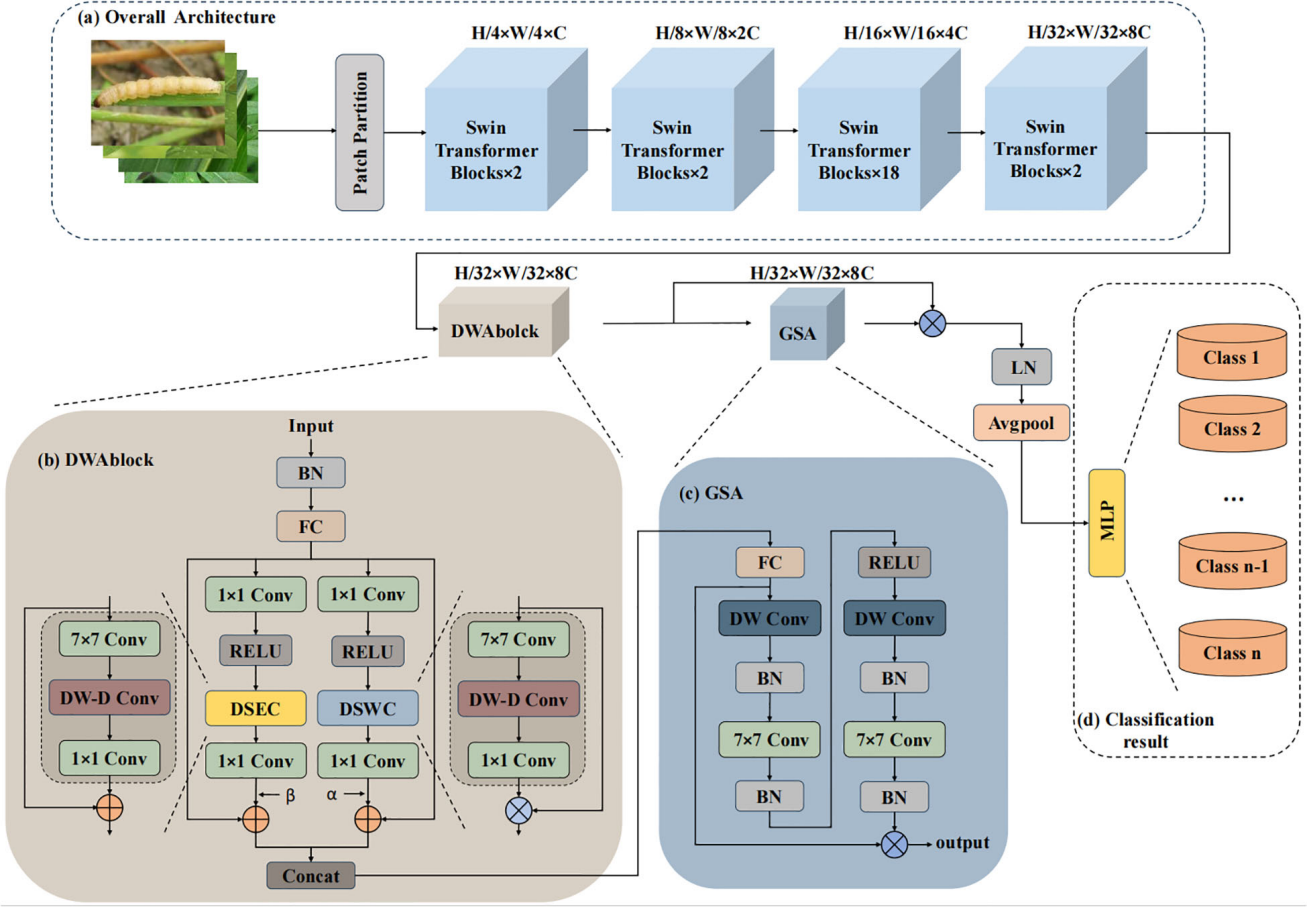
The overall architecture of Swin-AARNet. Swin-AARNet consists of three parts; **(a)** The first part is a backbone network composed of multiple Swin Transformer Blocks, **(b)** the second part is the depth-wise separable residual attention block DWAblock, **(c)** the last part is the global spatial attention GSA, and **(d)** represents MLP (Multilayer Perceptron), generating the final output result (specific category).

The overall architecture of Swin-AARNet, as illustrated in **Figure 2**, consists of three main components: the Swin backbone network, DWAblock, and GSA. Together, these elements form the Swin-AARNet network.

The backbone network is composed of multiple Swin Transformer blocks. It processes three-channel RGB insect images, extracting global feature information from the input images. The global features are then passed to the channel-local feature extractor, known as the DWAblock. After processing through the DWAblock, the feature images acquire three-channel self-attention information, which enhances the channel-specific features of the images.

Subsequently, the self-attention information is fed into the GSA module. The GSA processes this data to generate spatial information weights, which are then multiplied with the input self-attention features to produce the final enhanced global features.

Swin-AARNet demonstrates an advantage in extracting and enhancing global feature information when handling fine-grained insect images. This capability makes it particularly powerful for fine-grained insect recognition, enabling more accurate category prediction.

### 3.3 Depth-wise separable residual attention block

**Figure 3:**
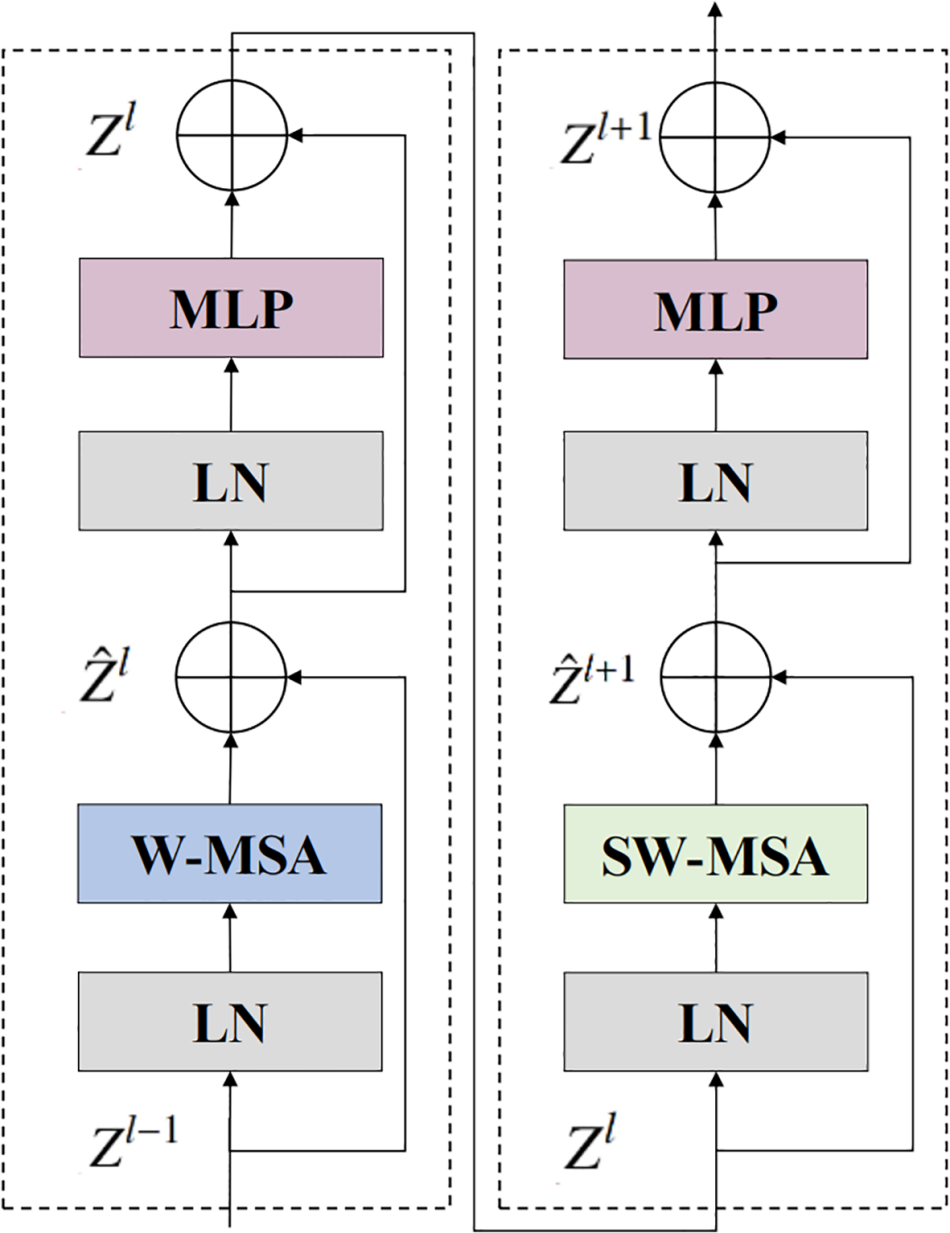
Detailed structure of the Swin Transformer block. W-MSA denotes window-based self-attention, and SW-MSA represents shifted window-based self-attention. ${\hat{Z}}^{l}$ and $Z^{l}$ denote the output feature map of the *i*th block after SW-MSA and MLP, respectively.


**Figure 3** illustrates the structure of the backbone network’s Swin Transformer block. The architectural formulation of the Swin Transformer block is described as follows (Equations 1–4):

(1) $${\hat{Z}}^{l}=W-MSA\left(LN\left(Z^{l-1}\right)\right)+Z^{l-1}$$

(2) $$Z^{l}=MLP\left(LN\left({\hat{Z}}^{l}\right)\right)+{\hat{Z}}^{l}$$

(3) $${\hat{Z}}^{l+1}=SW-MSA\left(LN\left(Z^{l}\right)\right)+Z^{l}$$

(4) $$Z^{l+1}=MLP\left(LN\left({\hat{Z}}^{l+1}\right)\right)+{\hat{Z}}^{l+1}$$

Here, $Z_{l-1}$ represents the result of the Swin Transformer block at layer l−1 , and W-MSA (Window Multi-head Self-Attention) is the window attention module that computes attention for $N=M^{2}$ patches within each window (Equation 5):


(5) $$\text{Attention}\left(Q_{k},K_{k},V_{k}\right)=\text{Softmax}\left(\frac{Q_{k}K_{k}^{T}}{\sqrt{d}}+B\right)V_{k}$$

Here $Q_{k},K_{k},V_{k}\in R^{N\times d}$ , where N denotes the number of patches in each window, and d is the feature dimension. B represents the relative position bias, which introduces learnable offsets for each position pair (i,j) within the local window. The SW-MSA (Shifted Window Multi-head Self-Attention) module cyclically shifts the feature map by ( $\left|\frac{M}{2}\right|$ , $\left|\frac{M}{2}\right|$ ) pixels to the right, followed by re-partitioning into new windows. The output $Z_{l+1}$ represents the result after applying both W-MSA and SW-MSA modules to $Z_{l-1}$ . By stacking multiple such Swin Transformer blocks, a relatively complete global feature representation can be obtained.

**Figure 4:**
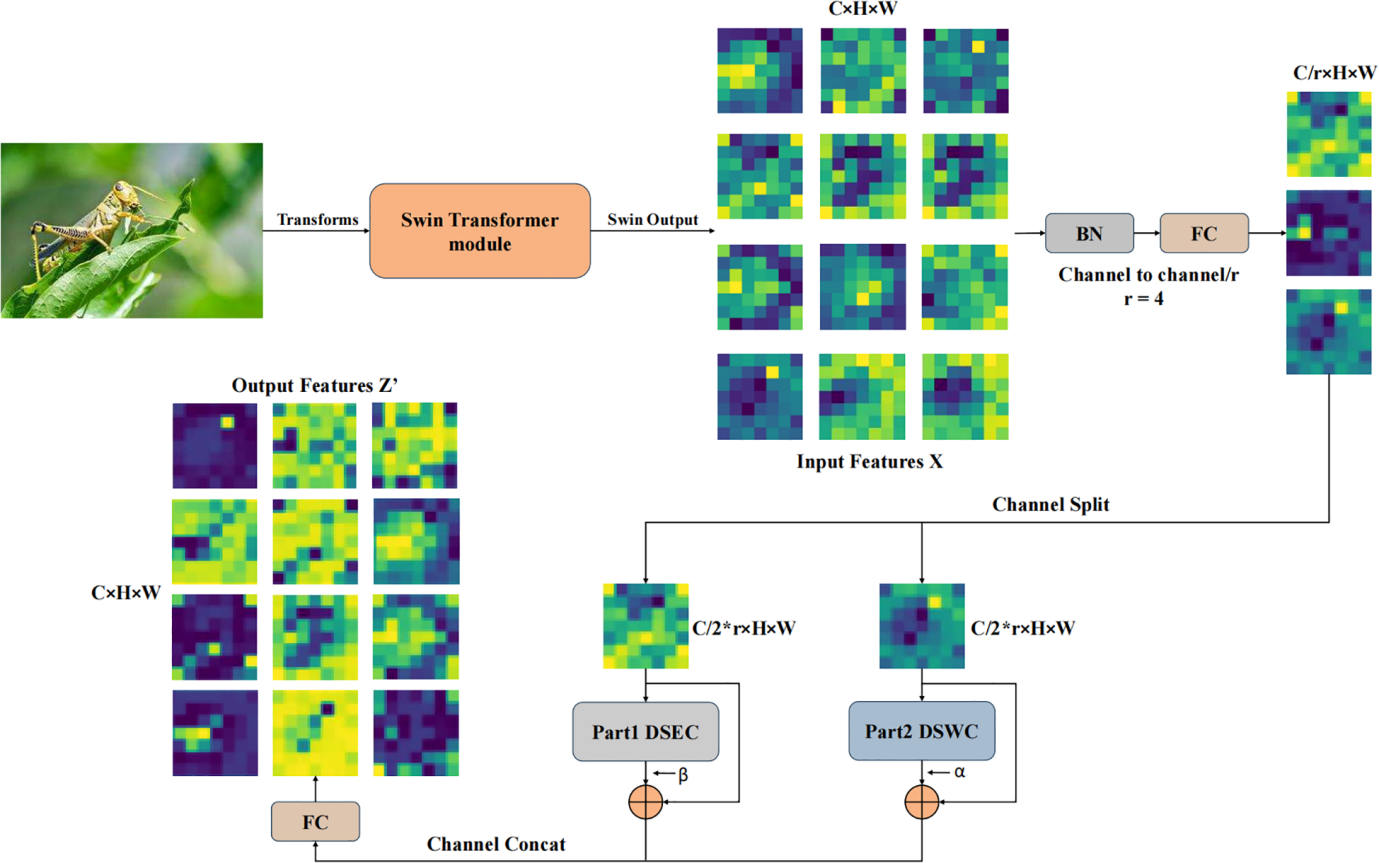
The structure of the DWA block can be described as follows: The input feature map has a channel number of *C*.After passing through a fully connected layer, the channel count is reduced by a factor of *r*.The *Split* operation divides the downsampled features along the channel dimension into two parts, with each part containing half of the original channel count. These two parts are then processed separately through *Part*1*DSEC* and *Part*2*DSWC* for channel feature extraction. The processed features are subsequently merged along the channel dimension using the *Concat* operation. Finally, a fully connected layer restores the channel count, producing the output features $Z^{\prime }$ .


However, relying solely on the W-MSA and SW-MSA self-attention mechanisms for pest recognition still exhibits certain limitations, particularly in capturing local discriminative features. To address this, a Depth-wise separable residual attention block isproposed, with its primary components illustrated in **Figure 4**. The formulation of the DWAblock is as follows (Equations 6–11):

(6) X=FC(BatchNorm(X))

(7) $$x_{c},x_{p}=\text{Split}\left(X\right)$$

(8) $$u_{c}=ReLU\left(W_{p}*x_{c}\right),\;u_{p}=ReLU\left(W_{p}*x_{p}\right)$$

(9) $${\tilde{u}}_{c}=W_{p}*\text{DSWC}\left(u_{c}\right),\;{\tilde{u}}_{p}=W_{p}*\text{DSEC}\left(u_{p}\right)$$

(10) $$Z=\text{Concat}\left(x_{c}+\alpha *{\tilde{u}}_{c},x_{p}+\beta *{\tilde{u}}_{p}\right)$$

(11) $$Z^{\prime }=FC\left(Z\right)$$

$W_{p}\in R^{H\times W\times C/2}$ denotes a 1×1 pointwise convolution. Here, the input X is divided along the channel dimension into two parts: $x_{c}$ and $x_{p}$ . These two parts are processed separately through the Depthwise Separable Weighted Convolution (DSWC) module and the Depthwise Separable Enhanced Convolution (DSEC) module. The outputs of the two branches are respectively assigned independent learnable weights *α* and β to dynamically adjust the contributions of the two branches and perform residual connections with the original branch inputs to promote model convergence and retain low-level feature information. The mathematical explanation for the DSWC and DSEC modules is as follows (Equations 12–17):

DSWC:


(12) $$v_{c}={\text{Conv}}_{7\times 7}\left(u_{c}\right)$$

(13) $$w_{c}=W_{p}*{\text{Depthwise}}_{d=3}\left(v_{c}\right)$$

(14) $${\tilde{m}}_{c}=w_{c}*u_{c}$$

DSEC:


(15) $$v_{p}={\text{Conv}}_{7\times 7}\left(u_{p}\right)$$

(16) $${\tilde{m}}_{p}=W_{p}*{\text{Depthwise}}_{d=3}\left(v_{p}\right)$$

(17) $${\tilde{m}}_{p}={\tilde{m}}_{p}+u_{p}$$

$u_{c}$ , $u_{p}\in R^{H\times W\times C/2}$ represent the outputs from the ReLU activation in the previous formulas, and ${\tilde{m}}_{c}$ , ${\tilde{m}}_{p}\in R^{H\times W\times C/2}$ be the output data from the DSWC and DSEC modules, respectively. According to the formulas, the DSWC and DSEC first apply a 7×7 grouped convolution to the feature map for depthwise convolution, enerating the attention base weight matrices $v_{c}$ and $v_{p}$ . This operation captures low-level visual features using a 7×7 local receptive field. Subsequently, the receptive field is expanded to 19×19 through a dilated convolution with adilation rate of 3 and a kernel size of 7×7 . The receptive field calculation formula is given by


(18) $$E_{K}=\left(K-1\right)\times D+1$$

where $E_{K}$ is the effective kernel size, **K** represents the actual kernel size, and **D** denotes the dilation rate (Equation 18). To maintain the same spatial dimensions of the feature map before and after the operation, the padding rate is set to 9. This allows the DWAblock to capture multi-scale global contextual information while preserving long-range spatial dependencies to some extent. Finally, $W_{p}\in {\mathbb{R}}^{H\times W\times C/2}$ is used to perform feature reorganization along the channel dimension, establishing cross-channel correlations and enhancing the local feature representation capability. Unlike DSEC, the DSWC modulates the original features **x** *_c_* using weights **w** *_c_* derived from a self-attention mechanism, thereby enhancing key features. In contrast, DSEC only performs a residual connection between the original features and the feature information. After the concatenation of DSWC and DSEC, both low-level details and semantic information are preserved. This not only dynamically enhances the important local features, improving model stability, but also retains the original features of the feature map to some extent, preventing the loss of fine-grained features. This forms a dual-path feature complementarity mechanism, enhancing the model’s expressive capability along the channel dimension.

### 3.4 Global Spatial Attention

In the previous subsection, the DWAblock was introduced in detail. The DWAblock integrates global features along the channel dimension. To further enhance the model’s representation capability and adaptability in the spatial dimension, this paper draws inspiration from the Global Attention Mechanism (GAM) (Liu et al., 2021a) and introduces structural improvements. GAM is designed to enhance the performance of deep neural networks by reducing information loss and amplifying global interactive representations. In its spatial attention submodule, GAM employs two convolutional layers to fuse spatial information. The formula for GAM is explained as follows (Equations 19, 20):


(19) $$\hat{A}={\text{Conv}}_{7\times 7}\left(Z^{\prime }\right)$$

(20) $$F=ReLU\left({\text{Conv}}_{7\times 7}\left(\hat{A}\right)\right)$$

**F** represents the output feature map from the GAM spatial attention module, while **Z** ^′^ denotes the output features from the DWAblock layer. The GAM’s spatial attention mechanism employs two 7×7 convolutional layers, incorporating a ReLU activation function between them to preserve the model’s non-linear characteristics. In particular, each convolutional layer is followed by Batch Normalization to stabilize the training process and accelerate convergence. However, this cross-dimensional spatial attention mechanism merges all channel spaces, effectively capturing complex inter-channel relationships but lacking the capacity to extract local features from each individual channel. This limitation results in an unintentional blending of spatial feature information.

**Figure 5:**
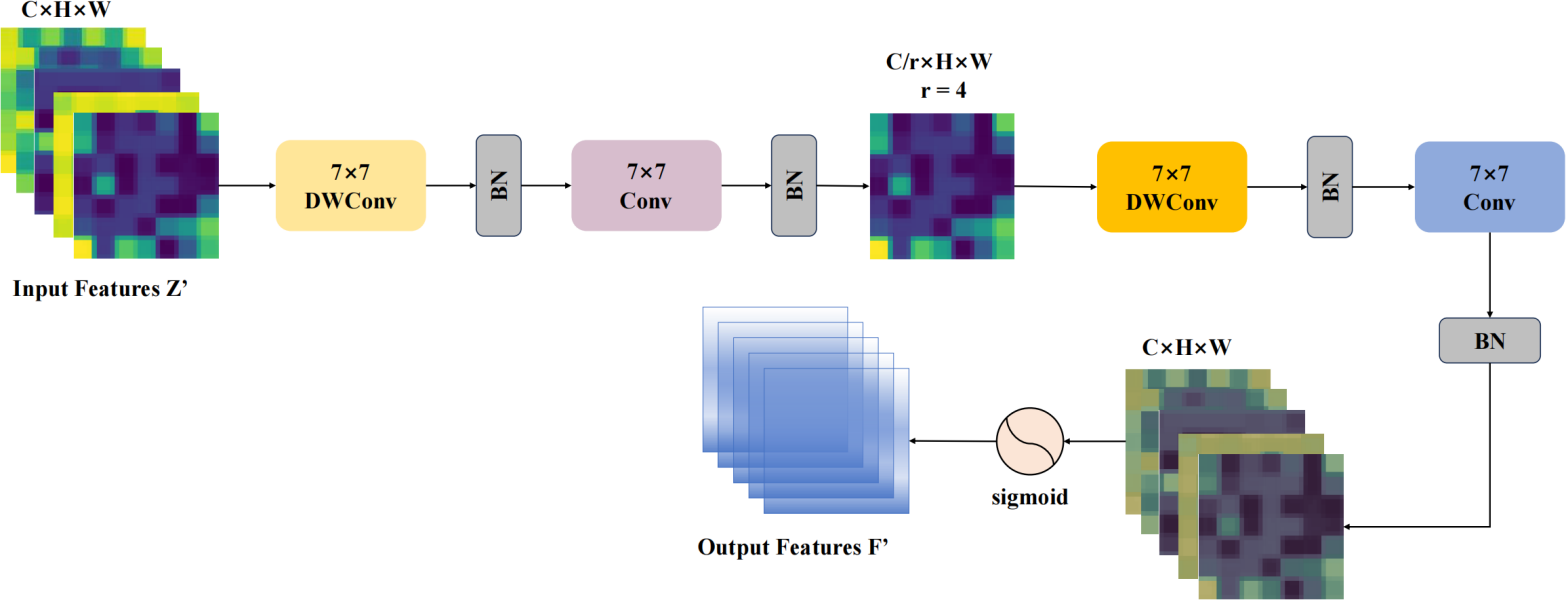
The structure of the GSA module is as follows: to effectively capture spatial information, the GSA submodule utilizes two depthwise convolutions along with two standard convolutions for spatial information fusion. Furthermore, the same reduction ratio *r* used in the channel attention submodule is applied here to maintain consistent feature scaling.


To address these issues, this study introduces a modification to the GAM’s spatial attention module, which we refer to as Global Spatial Attention. The architecture of the improved GSA model is illustrated in **Figure 5**, with the revised formulation provided below:

(21) $$Z={\text{Depthwise}}_{7\times 7}\left(Z^{\prime }\right)$$

(22) $$\hat{A}={\text{Conv}}_{7\times 7}\left(Z\right)$$

(23) $$F={\text{Depthwise}}_{7\times 7}\left(ReLU\left(\hat{A}\right)\right)$$

(24) $$F^{\prime }={\text{Conv}}_{7\times 7}\left(F\right)$$

Given an input feature map $Z^{\prime }\in {\mathbb{R}}^{H\times W\times C}$ , the GSA module enhances spatial features progressively through four convolutional operations. First, a 7×7 depthwise convolution is applied to extract initial deep features while preserving channel-wise independence, as shown in Equation 21. The output of this operation is denoted as **Z**. Next, channel reduction is performed to model more effective inter-channel dependencies and emphasize important channels, enabling each group of features to be processed with greater precision. The result of this channel reduction in the original spatial attention structure is denoted as $\hat{A}$ in Equation 22. ReLU activation is then applied to the reduced features, followed by another depthwise convolution to enhance local details and improve fine-grained feature representations (Equation 23). Finally, a 7×7 convolution is used to restore the original channel dimension, completing the multi-scale feature fusion. The output feature map of GSA, denoted as $F^{\prime }$ , thus benefits from stronger spatial feature extraction capabilities (Equation 24), enabling the model to capture finer-grained spatial information across different scales.

The improved GAM spatial attention incorporates depthwise convolution between the two standard convolution layers, effectively enhancing the extraction of spatial features. This approach not only reduces the computational overhead of the original spatial attention mechanism but also preserves channel independence, thereby enhancing the model’s ability to capture local spatial features. The effectiveness and practicality of the DWAblock and GSA for fine-grained pest image recognition are validated in the experimental section below. Additionally, the effectiveness of these components is demonstrated through various evaluation metrics when integrated with the Swin Transformer.

## 4 Results

### 4.1 Datasets

This experiment assesses the performance of Swin-AARNet on three datasets: IP102 (Insect Pest Dataset 102) (Wu et al., 2019) and CPB (Citrus Pest Benchmark) (Bollis et al., 2020) and Li (Li et al., 2020).

IP102 Dataset: The IP102 dataset is a pest dataset comprising 75,222 images, divided into 45,095 images for training, 22,619 images for testing, and 7,508 images for validation. The dataset includes 102 subcategories, 2 superclasses, and 8 major classes based on instance distribution. The hierarchical labeling system of IP102 categorizes the 102 subcategories based on the primary crops affected by the pests, resulting in 8 major classes, such as rice and maize, and further classifies them into 2 superclasses: field crops and economic crops. The imbalanced distribution across different levels poses challenges for learning from imbalanced data and using hierarchical labels.

The severity of pest damage to crops varies across different life stages, so the dataset includes images of all stages—eggs, larvae, pupae, and adults. For classification models, categorizing these subcategories into the same class presents a challenge, as it is difficult to extract distinctive features that clearly differentiate them. In addition to biodiversity, the imbalance in data distribution cannot be overlooked. As shown in **Figure 1**, the dataset exhibits a certain degree of imbalance across most subcategories in IP102. The dataset has been officially split into training, testing, and validation sets, eliminating the need for manual data splitting.

**Figure 6:**
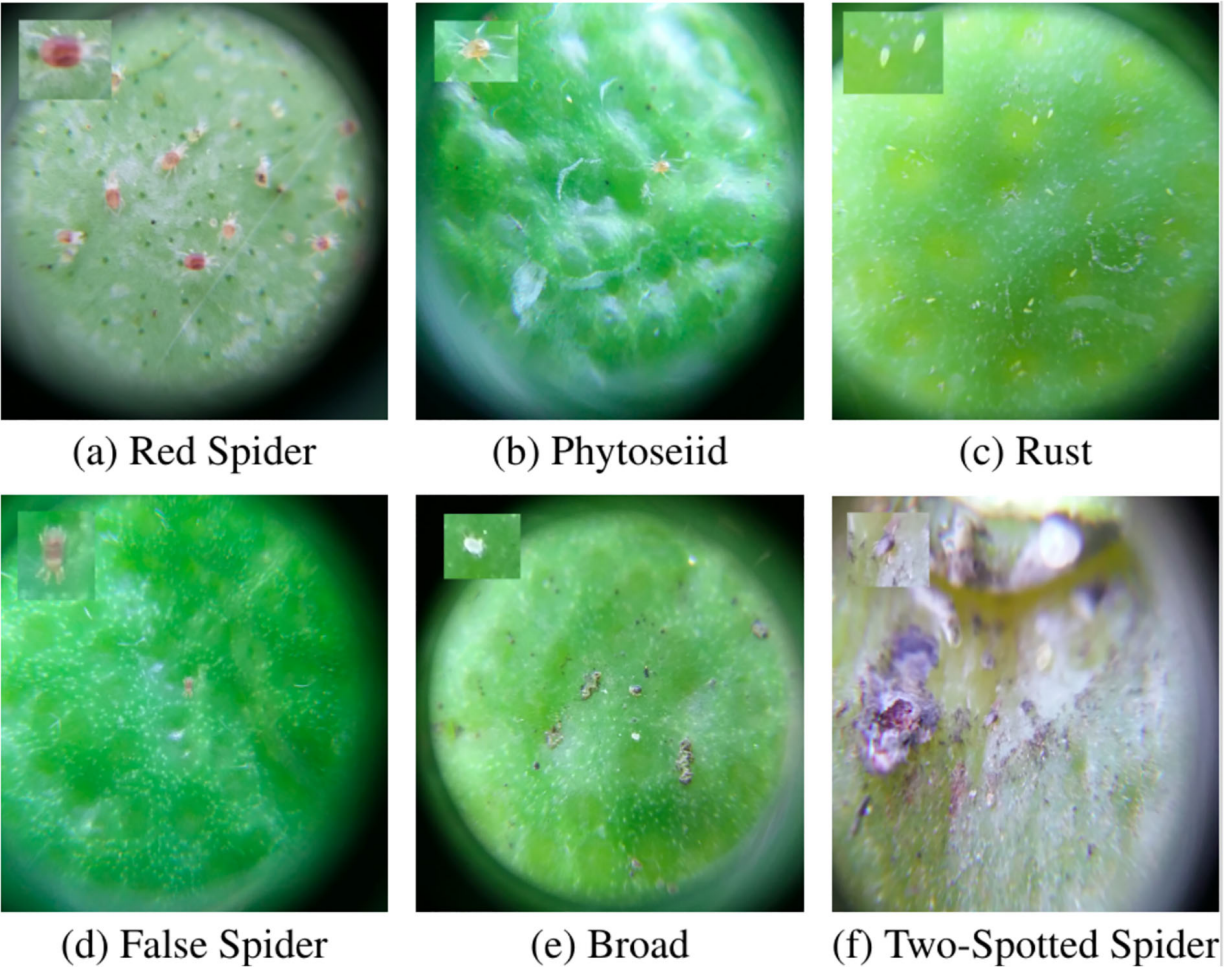
Example of CPB dataset. The mites are highlighted on the upper-left side of the images. Here, six types of frontal images are represented respectively: **(a)** Red Spider, **(b)** Phytoseiid, **(c)** Rust, **(d)** False Spider, **(e)** Broad, and **(f)** Two-Spotted Spider.

To further validate the effectiveness of Swin-AARNet, we conducted additional experiments on the CPB dataset. **Figure 6** illustrates sample images of mite pests from the CPB dataset. The CPB dataset contains 10,816 multi-class images divided into 7 categories, such as (i) 1,902 images of red spider mites (Bankeutetranychus, Mexicanus Tetranychus), which are the largest among other species and cause yellow symptoms on leaves and fruits; (ii) 1,426 images of predatory mites (Euseius citrifolius, Iphiseiodes zuluagai), which help control other mites; (iii) 1,386 images of rust mites (Phyllocoptruta oleivora), responsible for rust symptoms and significant crop losses; (iv) 1,750 images of false spider mites, carriers of the leprosis virus (Brevipalpus phoenicis); (v) 806 images of broad mites (Polyphagotarsonemus latus), which form white caps on fruits; (vi) 696 images of two-spotted spider mites, which, though not causing significant crop damage, are visible in the field; (vii) 3,455 negative images.

Due to the small size of the mites, there is substantial inter-class variation, and the dataset curators are currently revising multi-class labels. For this study, the CPB dataset was restructured into positive and negative classes of 1200×1200 pixel images. Specifically, categories (i) to (vi) (comprising 7,966 images) were combined as positive images, while the 3,455 negative images were treated as a separate class. During the experiments, the image set was divided into three groups—training, validation, and test—following the official dataset split of 60%, 20%, and 20%, respectively, yielding 6,380, 2,239, and 2,197 images.

**Figure 7:**
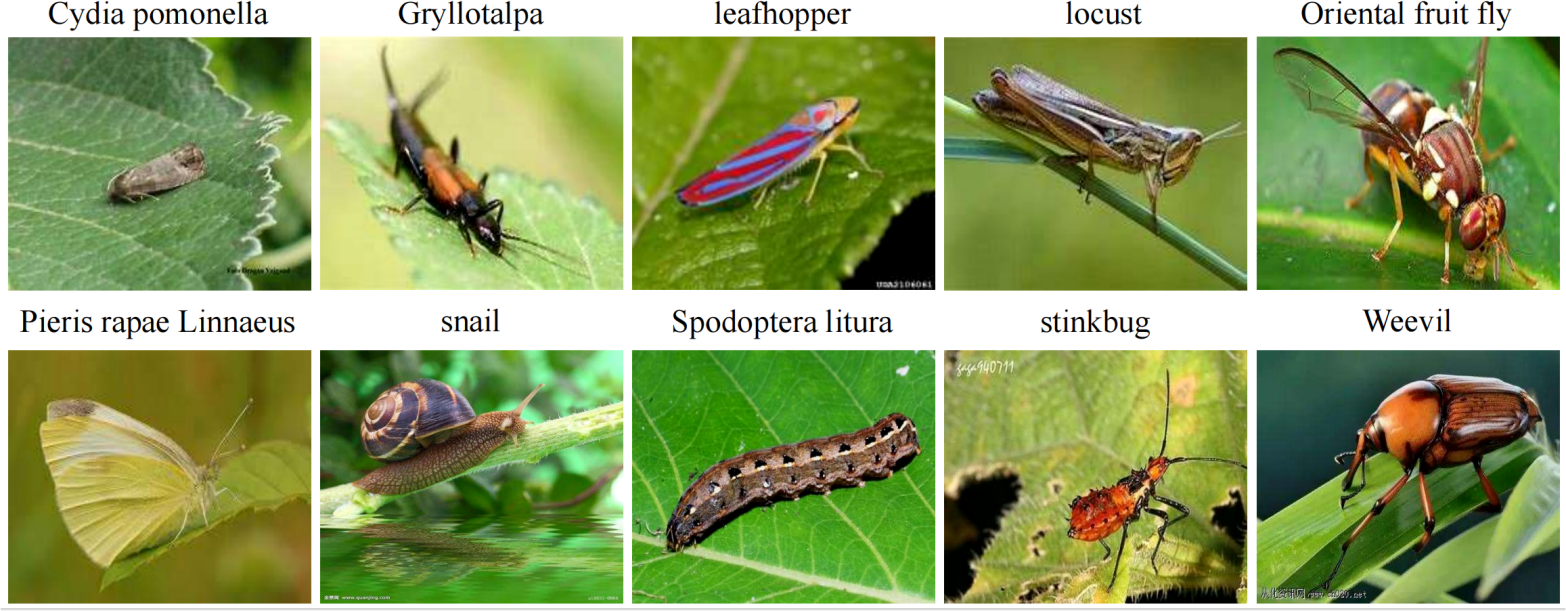
Example of Li.

To further validate the generalization capability of Swin-AARNet, we conducted additional experiments on the Li dataset. **Figure 7** displays a subset of samples from the Li dataset, which comprises 5,629 images covering 10 common pest categories: Gryllotalpa, Leafhopper, locust, Oriental fruit fly, Pieris rapae Linnaeus, Snail, Spodoptera litura, Stinkbug, Cydia pomonella, and Weevil. These pests primarily cause severe damage to staple crops such as rice, wheat, corn, soybeans, and sweet potatoes. In this study, we employed the relatively small Li dataset to assess Swin-AARNet’s generalization ability, further demonstrating its effectiveness in common pest infestation scenarios. During the experiments, the dataset was partitioned into three subsets—training, validation, and test—following the official dataset split of 80%, 10%, and 10%, respectively, resulting in 4,503, 563, and 563 images.

To ensure the fairness and accuracy of the experimental results, all experiments were conducted an NVIDIA GeForce RTX 3090 GPU with 24 GB of memory. The pre-trained weights of the Swin Transformer model, originally trained on the ImageNet-22K dataset, were used as the initial weights for these experiments. The detailed configuration of the experimental parameters is provided in **Table 2**. We applied random cropping to resize input images to 224 × 224 for data augmentation. The model was trained for 200 epochs with a batch size of 64. The AdamW optimizer was utilized with an initial learning rate of 5×10^−5^, and a cosine annealing schedule was adopted for dynamic learning rate adjustment. A weight decay of 0.01 was applied to mitigate overfitting. A learning rate warm-up strategy was employed during the first 5 epochs to enhance training stability and accelerate convergence. All model parameters were initialized randomly. Furthermore, all experiments were conducted in a Python 3.7 and PyTorch 1.13.0 environment to ensure the stability and reproducibility of the training and evaluation processes.

**Table 2:** Detailed settings for pre-training, fine-tuning, and transfer learning.

Task	Model	Datasets	Input	Epochs	Batch size	Optimizer	LR	LR decay	Weight decay	Warmup epochs
Pretrain	Swin Transformer	ImageNet-22K	224	90	4096	AdamW	1.00E-03	Cosine	0.01	5
Finetune	Swin-AARNet (ours)	IP102	224	200	64	AdamW	5.00E-5	Cosine	0.01	5
Finetune	Swin-AARNet (ours)	CPB	1200	200	64	AdamW	5.00E-5	Cosine	0.01	5
Finetune	Swin-AARNet (ours)	Li	224	200	64	AdamW	5.00E-5	Cosine	0.01	5

### 4.2 Ablation investigations

**Figure 8:**
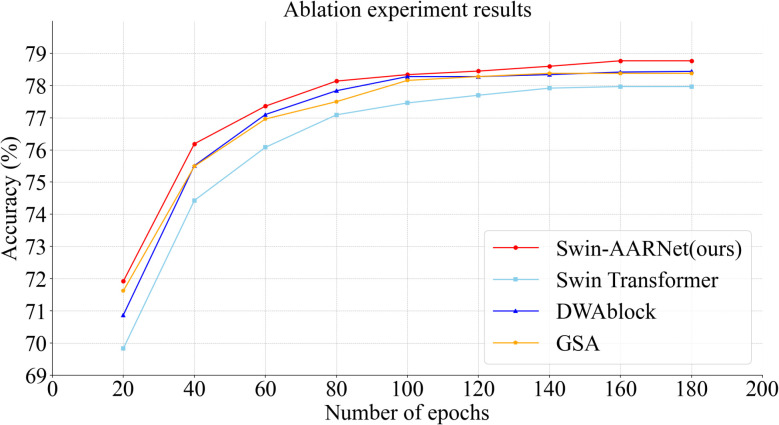
Figure of ablation experiment results on IP102. To enhance the visualization of the experimental results, we selected accuracy values at intervals of every 20 epochs within the range of 20 to 180 epochs. The results indicate that each component of Swin-AARNet is effective, demonstrating the robustness of its design.

This section provides a detailed explanation of the ablation experiments conducted on the IP102 and CPB datasets using Swin-AARNet, as shown in **Table 3**. The first and second columns indicate whether the corresponding module is used, with a tick symbol representing usage and a cross symbol indicating non-usage. In terms of training strategy, we adopt a 200-epoch scheme. As shown in **Figure 8**, although the validation accuracy plateaus after 100 epochs, continuing the training to 200 epochs still yields approximately a 0.5% improvement in Top-1 accuracy. This phenomenon suggests that after the model has completed global feature extraction, additional training epochs are still required to enhance the discrimination of fine-grained features. This characteristic is closely related to the structural appearance of pests, highlighting the necessity of extended training for fine-grained feature learning. Regarding the specific experimental design, First, we set the standard Swin Transformer, without any additional enhancements, as the baseline for reference and comparison. On this baseline, we then added the DWAblock, which resulted in an increase in accuracy to 78.44% and 81.46%, demonstrating that DWAblock can effectively extract global channel features.

**Table 3:** Ablation experiment of Swin-AARNet (Ours) on the IP102 and CPB Datasets. Among them, "✔" indicates that the module is included, and "✘" indicates that the module is not included.

Methods		IP102				CPB			
DWAblock	GSA	Params (M)	Epochs	PM (MiB)	Acc. (%)	Params (M)	Epochs	PM (MiB)	Acc. (%)
✘	✘	195.15	200	17760	77.97	195.00	200	17748	81.23
✔	✘	196.46	200	17780	78.44	196.31	200	17767	81.46
✘	✔	253.06	200	20517	78.38	252.90	200	20524	81.72
✔	✔	254.37	200	20593	78.77	254.22	200	20582	82.17

Here, PM stands for Peak memory.

Next, we integrated the GSA into the baseline, leading to an accuracy improvement to 78.38% and 81.72%, indicating that GSA enhances the integration of spatial information. Finally, by incorporating both the DWAblock and GSA into the baseline simultaneously, the accuracy improved significantly, reaching optimal levels of 78.77% and 82.17%.

**Figure 8** provides a visual representation of the changes and trends in the training process of the baseline Swin Transformer, highlighting the differences between each component of Swin-AARNet and the baseline. This visual evidence supports the effectiveness of each component in Swin-AARNet and validates the rationale behind their combination. These results not only demonstrate the theoretical feasibility of SwinAARNet but also confirm its efficacy through experimental validation, showing that Swin-AARNet achieves commendable results and highlights its advantages in handling insect image recognition tasks.

### 4.3 Comparison to the SOTA

To validate the rationale and accuracy of the proposed method, we compared our approach with the baseline models on the IP102 and CPB datasets and Li, as well as with seven state-of-the-art self-supervised classification methods. As shown in **Tables 4**, **5**, we present the model parameters, computational workload, peak memory usage, and accuracy. The peak memory usage was measured on a single NVIDIA 3090 GPU, with all experiments conducted using a fixed batch size of 64 and accelerated by mixedprecision training. The seven state-of-the-art self-supervised classification methods are (1)ConvNeXt (Liu et al., 2022c) (2)Cswin (Dong et al., 2022) (3)Data-efficient image Transformers (DeiT, DeiTv2) (Touvron et al., 2021, 2022) (4)Rethinking Model Scaling for Convolutional Neural Networks (EfficientNet) (Tan and Le, 2019) (5)Swin Transformer(SwinT, Swinv2) (Liu et al., 2021b, 2022b) (6)High Performance GPU-Dedicated Architecture (TResNet) (Ridnik et al., 2021) (7)Revisiting the Design of Spatial Attention in Vision Transformers(Twins) (Chu et al., 2021).

**Table 4:** Swin-AARNet (Ours) compares results with other models on the IP102 and CPB datasets.

Methods	Model size (MiB)	Epochs	Resolution	IP102			CPB		
				Params (M)	PM (MiB)	Acc. (%)	Params (M)	PM (MiB)	Acc. (%)
Convnext-B	338	200	224×224	87.67	9784	75.09	87.57	9782	80.83
Convnext-L	754	200	224×224	196.39	15961	74.97	196.23	15959	81.77
ResNet-50	97.7	200	224×224	23.72	5539	72.64	23.51	5537	75.20
ResNet-101	170	200	224×224	42.71	6972	73.23	42.50	6971	77.08
Cswin-B	295	200	224×224	76.69	12519	76.15	76.69	12517	81.46
Cswin-L	661	200	224×224	172.23	20329	78.41	–	–	–
DeiT-T	21.86	200	224×224	5.54	1742	68.61	5.54	1742	77.17
DeiT-S	84.16	200	224×224	21.74	3650	74.27	21.67	3646	79.04
DeiT-B	330.28	200	224×224	85.88	8124	75.80	85.80	8118	77.79
DeiTv2-S	84.21	200	224×224	21.71	3861	74.35	21.71	3860	78.95
DeiTv2-M	148.26	200	224×224	38.39	5339	75.52	38.39	5340	79.85
DeiTv2-B	330.35	200	224×224	85.89	8542	76.33	85.82	8537	79.31
DeiTv2-L	1161.8	200	224×224	303.45	15219	77.64	–	–	–
Efficient-b7	254	200	224×224	64.05	21333	73.11	63.79	19001	79.40
Tresnet-L	214	200	224×224	53.80	9820	75.95	53.56	9817	77.03
Tresnet-XL	299	200	224×224	76.05	12076	76.23	75.78	12075	78.73
ViT-B	330	200	224×224	85.88	8117	74.99	85.80	8115	77.26
ViT-L	1161.6	200	224×224	303.41	22837	75.28	–	–	–
Twins-B	167	200	224×224	43.37	7876	75.47	43.37	7877	80.56
Twins-L	232	200	224×224	60.53	10867	75.76	60.53	10850	81.19
Swin-T	109	200	224×224	27.60	4933	74.57	27.52	4930	80.47
Swin-S	190	200	224×224	48.92	7891	75.45	48.84	7892	80.25
Swin-B	336	200	224×224	86.85	10984	74.72	86.75	10980	80.65
Swin-L	751.35	200	224×224	195.15	17760	77.97	195.00	17748	81.23
Swin-AARNet(ours)	751.35	200	224×224	254.37	20593	78.77	254.22	20582	82.17

During the experiments, we utilized a single GPU for model training and inference. Due to memory limitations, ‘-’ denotes instances where an out-of-memory error occurred during training. To ensure consistency across the experimental setup, those experiments affected by memory overflow were excluded from the analysis.

**Table 5:** Comparison of Swin-AARNet (Ours) with other models on the Li dataset.

Methods	Model size (MiB)	Epochs	Resolution	Li			
				Params (M)	PM (MiB)	Acc. (%)	F1. (%)
Convnext-B	338	200	224×224	87.67	9784	98.30	98.05
Convnext-L	754	200	224×224	196.24	15959	98.97	98.82
ResNet-50	97.7	200	224×224	23.52	5537	98.28	98.21
ResNet-101	170	200	224×224	42.52	6971	98.97	98.88
Cswin-B	295	200	224×224	76.69	12518	98.97	98.76
Cswin-L	661	200	224×224	172.12	20329	99.14	98.99
DeiT-T	21.86	200	224×224	5.53	1742	97.08	96.83
DeiT-S	84.16	200	224×224	21.70	3648	97.77	97.41
DeiT-B	330.28	200	224×224	85.81	8120	97.94	97.73
DeiTv2-S	84.21	200	224×224	21.68	3861	97.94	97.62
DeiTv2-M	148.26	200	224×224	38.34	5341	98.28	98.02
DeiTv2-B	330.35	200	224×224	85.82	8540	98.63	98.19
Efficient-b7	254	200	224×224	63.81	21332	96.74	96.45
Tresnet-L	214	200	224×224	53.58	9817	97.94	97.66
Tresnet-XL	299	200	224×224	75.81	12073	98.63	97.97
ViT-B	330	200	224×224	85.80	8113	97.77	97.35
ViT-L	1161.6	200	224×224	303.31	22850	98.97	98.78
Twins-B	167	200	224×224	43.32	7876	98.63	98.50
Twins-L	232	200	224×224	60.48	10867	98.97	98.80
Swin-T	109	200	224×224	27.53	4932	98.11	97.81
Swin-S	190	200	224×224	48.84	7896	98.45	98.21
Swin-B	336	200	224×224	86.75	10985	98.63	98.40
Swin-L	751.35	200	224×224	195.01	17754	98.97	98.84
Swin-AARNet(ours)	751.35	200	224×224	254.23	20358	99.48	99.37

During the experiments, all models were trained and inferred using a single GPU.

To validate the model’s generalization capability, we compared Swin-AARNet with the latest methods on the Li dataset. As shown in **Table 5**, Swin-AARNet achieved a classification accuracy of 99.48% and an F1-score of 99.37%, demonstrating superior generalization performance. The results confirm the model’s strong adaptability to diverse pest recognition scenarios.

From **Table 4**, it can be observed that the highest accuracy achieved by convolutional neural networks on the two datasets is 76.23% and 81.77%. Vision Transformers and their variants achieved a maximum accuracy of 77.64% and 79.31% across several subsets, while CSwin reached a peak accuracy of 79.85%. Swin-AARNet outperformed all, achieving the highest accuracy of 78.77% and 82.17% on both datasets, respectively. Compared to ResNet, Swin-AARNet not only focuses on local feature representations but also effectively captures global features from the feature maps while extracting local details. Compared to Vision Transformer and its variants, Swin-AARNet excels at extracting more detailed channel and spatial feature information, enabling it to capture subtle differences within insect images with greater accuracy. Additionally, Swin-AARNet results from the integration of Swin Transformer and an attention mechanism. Compared to conventional methods, Swin-AARNet effectively distinguishes between different insect categories and demonstrates strong generalization ability, maintaining high accuracy across various developmental stages of insects. However, compared to Cswin-B, Cswin-L, Twins-L, Convnext-L, and Swin-L, Swin-AARNet has the drawback of having a larger number of model parameters and higher peak memory usage. Therefore, in addition to accuracy, we also evaluate model performance using three commonly used image classification metrics. Precision (Pre), Recall (Rec) and F1 score (F1). As shown in **Table 6**, we present the results of the Swin-AARNet evaluation along with Cswin-B, Cswin-L, Twins-L, Convnext-L, and Swin-L across these four metrics. The results show that Swin-AARNet outperforms all these models in terms of Pre, Rec, and F1. This indicates that Swin-AARNet not only achieves high accuracy and excellent fitting but also exhibits strong generalization ability on unseen samples, allowing it to accurately infer data patterns and make correct predictions. Consequently, the model is well suited for handling insects with similar shapes but belonging to different categories.

**Table 6:** Six evaluation metrics of Cswin-B, Cswin-L, Twins-L, Convnext-L, Swin-L and Swin-AARNet (Ours) on IP102 and CPB datasets, ‘-’ denotes instances where an out-of-memory error occurred during training.

Methods	Epochs	Resolution	IP102				CPB			
			Acc. (%)	Pre. (%)	Rec. (%)	F1. (%)	Acc. (%)	Pre. (%)	Rec. (%)	F1. (%)
Cswin-B	200	224 × 224	76.15	71.20	67.89	68.39	81.46	78.51	77.12	77.74
Cswin-L	200	224 × 224	78.41	73.13	70.83	71.48	–	–	–	–
Twins-L	200	224 × 224	75.76	69.57	66.66	67.47	81.19	78.32	76.36	77.19
Convnext-L	200	224 × 224	74.97	68.82	65.65	66.10	81.77	79.15	76.48	77.55
Swin-L	200	224 × 224	77.97	71.77	70.38	70.69	81.23	78.53	76.03	77.05
Swin-AARNet (ours)	200	224 × 224	78.77	73.14	71.65	71.97	82.17	79.63	77.35	78.30

To ensure consistency across the experimental setup, those experiments affected by memory overflow were excluded from the analysis.

**Figure 9:**
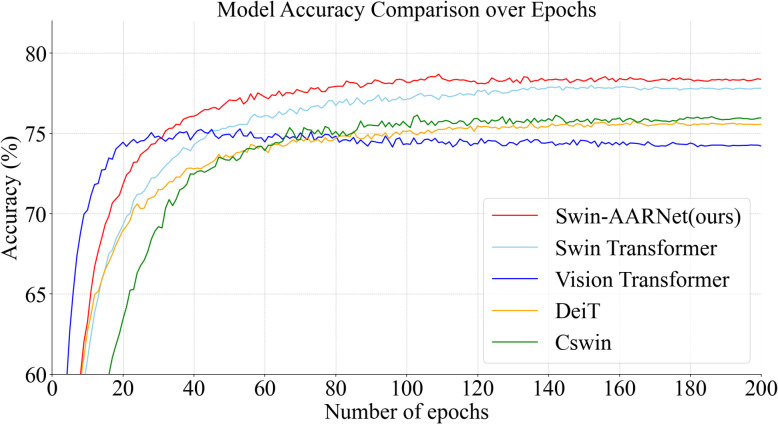
Comparison results between Swin-AARNet and other advanced methods.

**Figure 9** illustrates the accuracy trends of Swin-AARNet compared to several state-of-the-art classification methods on the IP102 insect image dataset. To ensure full convergence, each model was trained for 200 epochs. As shown, Swin-AARNet exhibits significant improvements in accuracy during both training and validation, with a relatively stable rate of increase. Swin-AARNet (red curve) consistently achieves higher accuracy throughout the training process and rapidly converges to a high accuracy level within the first 30 epochs, highlighting its advantages in stability and convergence speed. In contrast, other models, such as Vision Transformer (blue curve) and DeiT (orange curve), show a similar rapid initial rise but ultimately reach a slightly lower final accuracy than Swin-AARNet. These comparative experiments further demonstrate that the components of Swin-AARNet are not only theoretically feasible but also practically effective, yielding superior results on IP102, CPB, Li datasets.

To further validate the effectiveness of Swin-AARNet in fine-grained image recognition tasks, we conducted comparative experiments with several open-source fine-grained recognition methods: (1) APCNN (Ding et al., 2021); (2) FFM and MAM (Qian et al., 2025); and (3) Pest-ConFormer (a hybrid convolutional neural network and transformer-based model) (Fang et al., 2024). As shown in **Table 7**, Swin-AARNet achieves the best performance across all three benchmark datasets: IP102, CPB, and Li. The competitive performance of Swin-AARNet can be attributed to the following aspects: (1) Multi-scale feature fusion: Unlike the dual-path feature aggregation structure in Pest-ConFormer and the dual-path hierarchical design in AP-CNN, Swin-AARNet explicitly captures multi-scale contextual information through convolutional modules with different dilation rates in the DSWC and DSEC blocks. Furthermore, it introduces learnable parameters (*α* and *β*) to adaptively control the contribution of features from different scales. (2) Grouped attention mechanism: While Pest-ConFormer relies on standard Transformer-based self-attention and MAM and FAM adopts a hybrid attention module, Swin-AARNet separates the feature channels into two groups and applies a channel attention mechanism to each branch independently. This design enables more precise attention control and preserves computational efficiency. (3) Fine-grained feature extraction capability: AP-CNN depends on an ROI-guided optimization strategy to extract discriminative features. In contrast, Swin-AARNet performs feature decoupling via channel separation and employs large-kernel convolutions to capture broader local details. This eliminates the need for additional annotations (e.g., ROI-guided), enabling fine-grained feature extraction through architectural design alone.

**Table 7:** Comparison results of four fine-grained image recognition methods, AP-CNN, FFM and MAM, Pest-ConFormer, and Swin-AARNet on the IP102, CPB, and Li datasets.

Methods	Epochs	Backbone	Resolution	IP102		CPB		Li	
				PM (MiB)	Acc. (%)	PM (MiB)	Acc. (%)	PM (MiB)	Acc. (%)
AP-CNN	200	CNN	224×224	9032	71.24	8762	70.72	8764	97.82
FFM+MAM	200	CNN+Transformer	224×224	16782	75.74	16774	76.82	16772	98.77
Pest-ConFormer	200	CNN+Transformer	224×224	19446	75.51	18362	79.89	18544	98.45
Swin-AARNet(ours)	200	CNN+Transformer	224×224	20593	78.77	20582	82.17	20358	99.48

These results clearly demonstrate the superior discriminative power of Swin-AARNet for fine-grained pest classification, suggesting that it exhibits strong stability and generalization ability, with reduced sensitivity to dataset and environmental variations.

### 4.4 Effectiveness of Swin-AARNet

**Figure 10:**
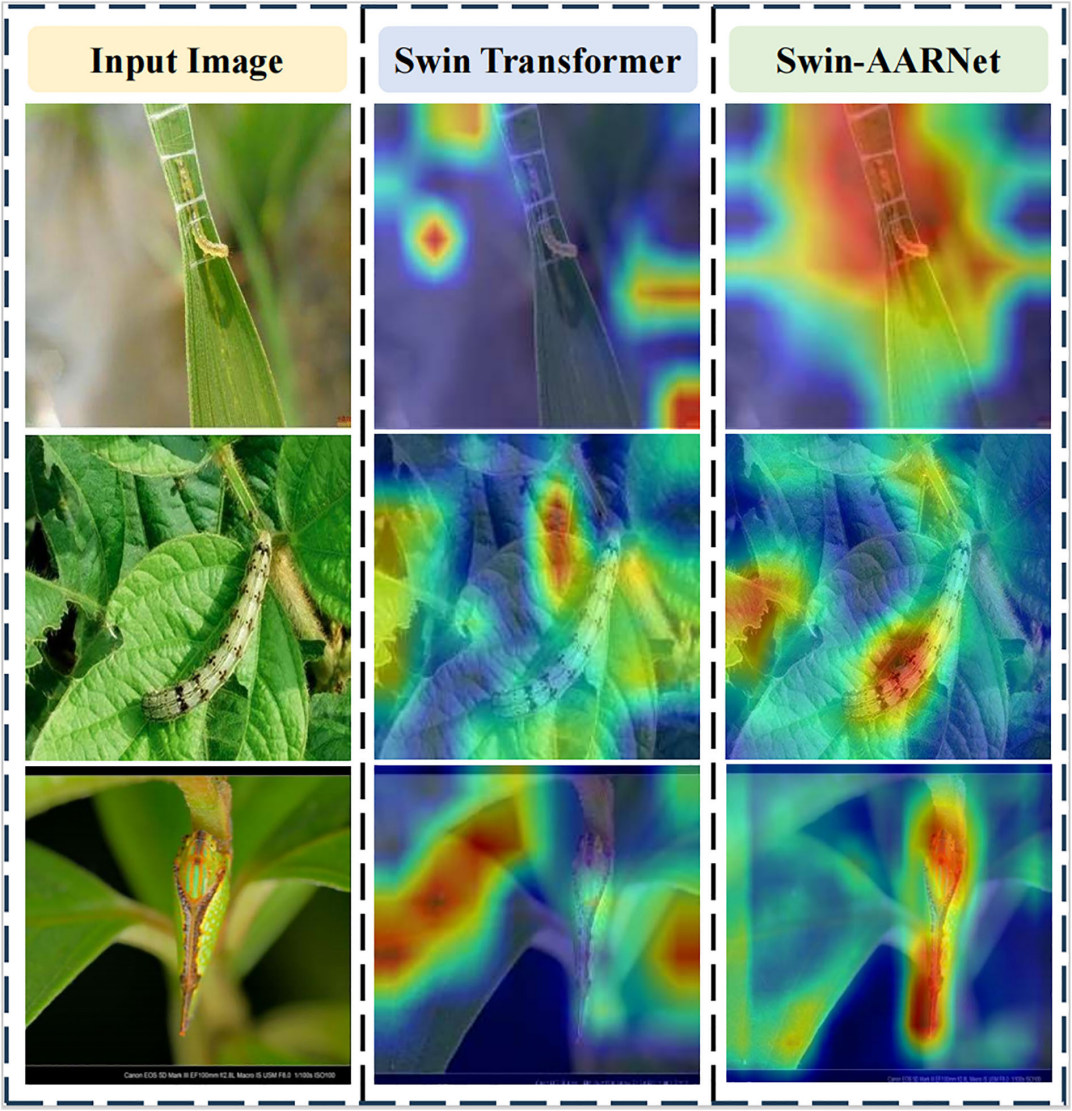
Grad-CAM images generated by the two modules. The first column shows three different pest raw images. The images in the second column are the Grad-CAM images acquired by Swin Transformer. The images in the third column are the Grad-CAM images obtained by Swi-AARNet. By Swin-AARNet, the model can extract pest features more comprehensively.

The proposed Swin-AARNet is designed to leverage fine-grained features in pest images for accurate classification of pests with similar appearances and under complex backgrounds. To further evaluate the effectiveness of Swin-AARNet, we employed the Gradient-weighted Class Activation Mapping (GradCAM) technique for visualization purposes. As shown in **Figure 10**, the red-highlighted regions in the Grad-CAM heatmaps represent the areas that the model focuses on. The results indicate that SwinTransformer tends to confuse background features in images with small pests, whereas Swin-AARNet effectively focuses on the majority of the pest body regions. By leveraging more precise fine-grained information, this method enables more accurate identification of pest shape and category.

**Figure 11:**
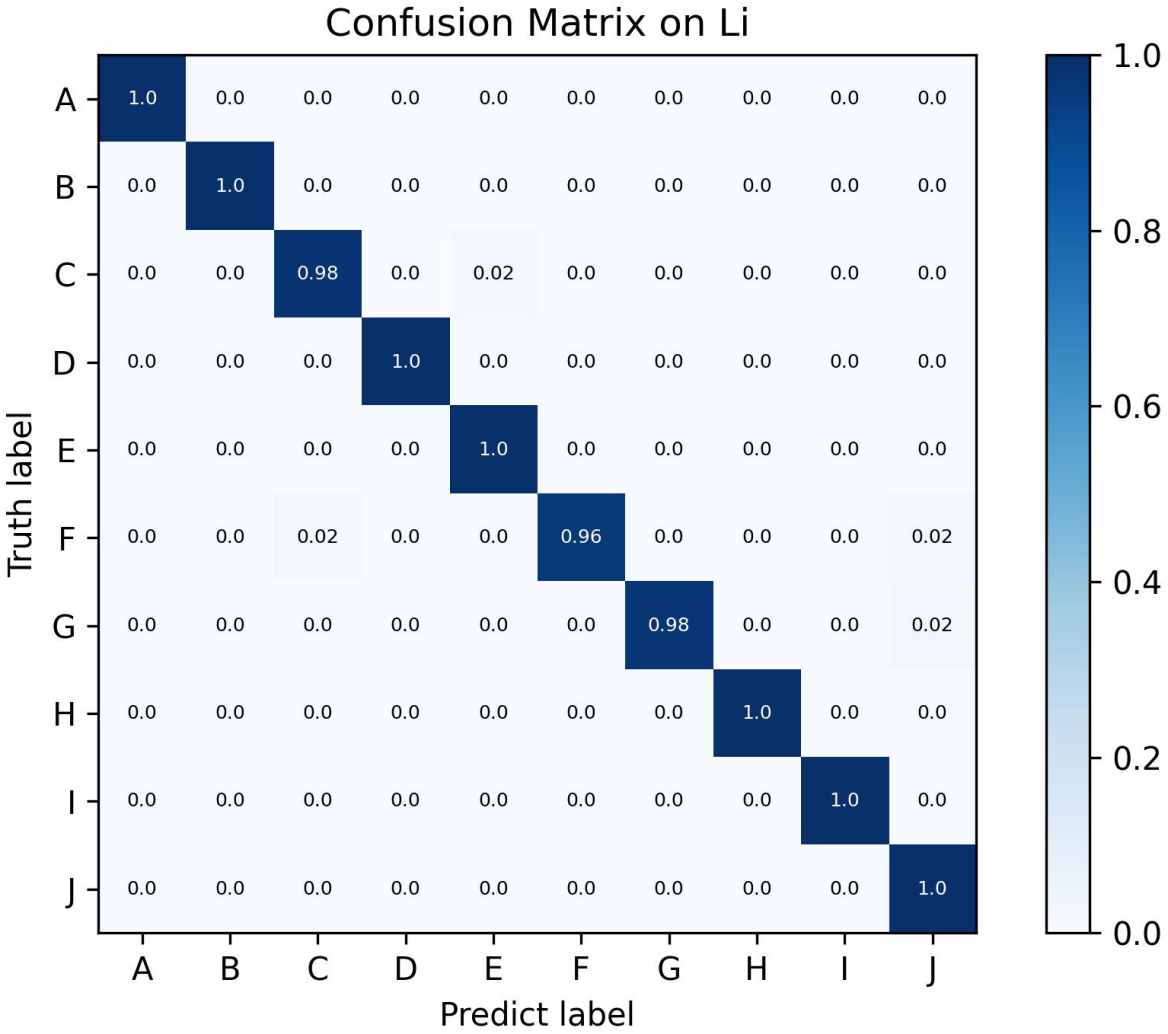
Confusion matrix on the Li dataset, where A stands for Gryllotalpa, B for Leafhopper, C for locust, D for Oriental fruit fly, E for Pieris rapae Linnaeus, F for Snail, G for Spodoptera litura, H for Stinkbug, I for Cydia pomonella and J for Weevil.

**Figure 11** presents the confusion matrix results of Swin-AARNet on the test set of the small-scale Li dataset, which includes 10 pest categories: Gryllotalpa, Leafhopper, locust, Oriental fruit fly, Pieris rapae Linnaeus, Snail, Spodoptera litura, Stinkbug, Cydia pomonella, and Weevil. The experimental results demonstrate that the model exhibits strong recognition performance across all 10 pest classes. Notably, Snail exhibits the highest misclassification rate, frequently being confused with Weevil, likely due to their morphological similarities (e.g., shell shape and size) and background interference in the images. In contrast, the model accurately recognized the remaining pest categories, further validating the effectiveness of Swin-AARNet in few-shot pest recognition tasks. This demonstrates its broad application potential. Our source code is available at: https://github.com/XiangWang888/Swin_ARRNet

## 5 Discussions

### 5.1 Model limitations

Although the proposed pest recognition method achieves state-of-the-art accuracy, we acknowledge several limitations, as outlined below.

First, as discussed in Section 3.4, the issue of data imbalance remains a major challenge. **Table 8** illustrates the hierarchical structure of the IP102 dataset, where Training/Validation/Test splits are denoted as Train/Val/Test, and IR represents the imbalance ratio across different class levels. Each sub-class is grouped into a super-class according to the primary crop it affects. These eight crop categories are further grouped into two higher-level super-classes: field crop and economic crop (FC and EC). As shown in **Table 8**, the IP102 dataset exhibits high imbalance ratio (IR), exceeding 91 in most super-class levels (Fernández et al., 2008). Such an imbalanced data distribution can lead to model bias, favoring classes with a larger number of training samples and affecting overall generalization.

**Table 8:** Train/validation/Test (denoted as Train/Val/Test) set split and imbalance ratio (IR) for the IP102 dataset at different class levels.

Super-Class		Class	Train	Val	Test	IR
FC	Rice	14	5043	843	2531	6.4
	Corn	13	8404	1399	4212	27.9
	Wheat	9	2048	340	1030	5.2
	Beet	8	2649	441	1330	15.4
	Alfalfa	13	6230	1037	3123	10.7
EC	Vitis	16	10525	1752	5274	74.8
	Citrus	19	4356	725	2192	17.6
	Mango	10	5840	971	2927	61.7
IP102	FC	57	24602	4098	12341	39.4
	EC	45	20721	3448	10393	80.8
IP102		102	45095	7508	22619	80.8

Class denotes the number of subclasses of the corresponding superclass. FC and EC denote field and cash crops.

Second, as shown in **Table 4**, Swin-AARNet achieves the best performance among all compared backbone models but seems to get the best performance at the cost of parameters. This is primarily due to the largescale architecture of its backbone network. The high parameter count presents challenges for real-world deployment on resource-constrained agricultural devices, particularly in terms of computational load and inference efficiency. Therefore, developing efficient model compression techniques—such as weight pruning, quantization, or knowledge distillation—is of great importance to promote the practical application of the proposed model in real-world agricultural scenarios.

### 5.2 Future work

With the advancement of global climate change and agricultural modernization, pest infestations have become one of the most critical challenges facing agricultural production worldwide. Severe outbreaks can significantly reduce crop yield and quality, leading to considerable economic losses and posing potential threats to food security. To address this issue, we propose a novel model, Swin-AARNet, which effectively tackles the challenges of multi-class and multi-morphological insect image recognition. Swin-AARNet shows great potential in supporting accurate pest identification and early warning, thereby contributing to improved pest management.

It is worth noting that although Swin-AARNet achieves the highest recognition accuracy in our experiments, its large number of parameters presents challenges for deployment on agricultural mobile devices. Consequently, future research will focus on model compression and optimization to enable lightweight deployment. At present, we have developed a desktop application that allows researchers to perform pest classification and query tasks, providing a practical solution for current agricultural needs.

**Figure 12:**
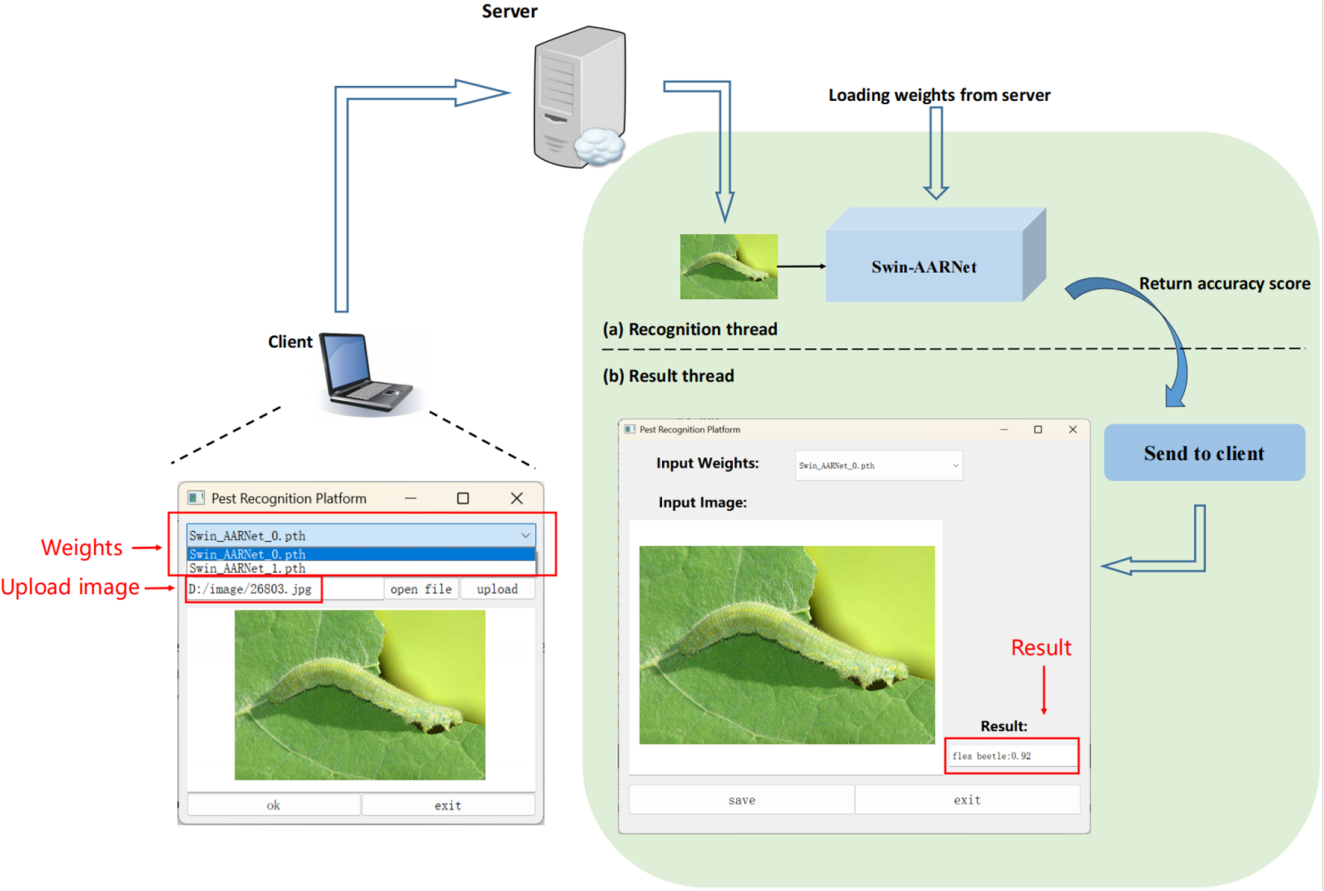
Pest recognition application.

As illustrated in **Figure 12**, the design of the desktop application is as follows: users capture pest images using mobile devices and upload them to a cloud server via the application. The server then invokes the trained recognition model to analyze and predict the pest category, returning the predicted class along with its confidence score to the application. The application supports result storage and management for later access and reference. In addition, the uploaded images are continuously incorporated into the training dataset on the server, enabling incremental learning and ongoing model optimization to improve adaptability to new environments and novel pest samples.

In practical pest image recognition tasks, several key factors must be taken into account. First, regarding image acquisition distance, modern devices such as smartphones and digital cameras commonly support 1080p resolution (1920×1080 pixels). Using collected images of snails as an example, we analyzed the relationship between the proportion of pest area within the image and the corresponding recognition accuracy. Experimental results show that when the pest occupies 3%–8% of the image area, the model achieves an accuracy of 97.60%. When the occupied area increases to 15%–35%, the accuracy improves significantly to 99.38%. However, when the pest area exceeds 50%, the recognition accuracy slightly decreases to 98.96%. These findings indicate that an image acquisition distance of approximately 10–30 cm (corresponding to a pest area proportion of 15%–35%) yields optimal model performance. If the distance is too great, the pest appears relatively small in the image, leading to insufficient detail, which may degrade recognition performance—especially under complex field conditions. Additionally, considering the dynamic nature of camera operation in real-world deployments, we evaluated the impact of camera motion on recognition accuracy. In slightly blurred images captured during camera movement, the model’s accuracy only declined by approximately 0.88%, demonstrating strong robustness. Regarding inference efficiency, the Swin-AARNet model was evaluated on an NVIDIA RTX 3090 platform. The theoretical inference time was approximately 0.003125 seconds per image, allowing for a throughput of about 320 images per second. In practical deployment scenarios—factoring in image loading and preprocessing overhead—the system achieves an overall processing frame rate of 25–30 FPS (Frames Per Second). This supports a camera movement speed of 1–1.5 meters per second while maintaining high recognition accuracy. For large-scale pest detection scenarios, efficiency can be further enhanced through strategies such as frame skipping and inter-frame feature fusion. In conjunction with high-resolution cameras and sliding window techniques, this allows for high-precision recognition across large image regions. It is also worth noting that recognition conditions vary by pest type. For relatively static pests such as snails, image acquisition is comparatively straightforward. However, for more active pests, high-frame-rate cameras are required to improve capture success rates and ensure accurate recognition by the model.

Furthermore, future studies in pest image recognition can be expanded to larger and more diverse datasets to further evaluate the model’s robustness under complex real-world conditions, such as variations in lighting, image resolution, and seasonal differences. These efforts will lay a solid foundation for the development of a more comprehensive and practical pest image classification system, ultimately enhancing crop protection and food security.

### 5.3 Conclusion

Pest image recognition presents several challenges, such as uneven inter-class variation and significant morphological differences across developmental stages. Some pest species exhibit extremely subtle interclass distinctions, while others undergo dramatic morphological changes throughout their life cycles. To address these issues, we propose a novel insect image recognition method, namely the Swin-AARNet: a deep residual attention network composed of a Swin Transformer backbone and two attention modules.

Our approach consists of several key components. The first is the DWAblock, which extracts channel-wise features from the feature maps. It not only integrates local feature information but also enhances the model’s ability to capture global context. The second component, GSA, is a spatial enhancement module introduced in this work. Built upon the DWAblock, GSA integrates spatial information across feature maps and captures global spatial features within individual channels, further improving the model’s capability to extract localized spatial details.

In the experimental section, we conduct extensive ablation studies, comparative experiments, and validation tests. Compared to state-of-the-art self-supervised methods, our proposed model achieves superior accuracy on public benchmark datasets IP102 and CPB and Li. The results demonstrate that Swin-AARNet not only fits well to the data but also exhibits strong generalization capabilities. These findings validate the effectiveness and rationality of the individual components of Swin-AARNet. In addition, we have preliminarily developed a desktop application to demonstrate the practical application of the proposed recognition method. In future work, we plan to further extend our efforts to mobile application development, aiming to provide more accessible and convenient solutions for agricultural pest identification.

## Data Availability

The datasets presented in this study can be found in online repositories. The data presented in this study are openly available in the IP102 dataset at https://github.com/xpwu95/IP102,The data presented in this study are openly available in Citrus Pest Benchmark at https://github.com/edsonbollis/Citrus-Pest-Benchmark. The data presented in this study are openly available in Li at https://doi.org/10.1016/j.compag.2019.105174.
